# First evidence for backcrossing of F_1_ hybrids in *Acropora* corals under sperm competition

**DOI:** 10.1038/s41598-022-08989-1

**Published:** 2022-03-30

**Authors:** Seiya Kitanobo, Kenji Iwao, Hironobu Fukami, Naoko Isomura, Masaya Morita

**Affiliations:** 1 Tropical Biosphere Research Center, Sesoko Station, University of the Ryukyus, Sesoko, Motobu, Okinawa 905-0227 Japan; 2 Akajima Marine Science Laboratory, Zamami, Okinawa 901-3311 Japan; 3 Department of Marine Biology and Environmental Science, Faculty of Agriculture, University of Miyazaki, Miyazaki, Japan; 4 Department of Bioresources Engineering, National Institute of Technology, Okinawa College, 905 Henoko, Nago, Okinawa 905-2192 Japan

**Keywords:** Evolution, Zoology

## Abstract

*Acropora* is a species-rich genus of reef-building corals with highly diverse morphologies. Hybridization among intercrossing species potentially influences species diversity within *Acropora*. However, the mechanisms that allow hybridization/backcrossing remain unknown. Although we tested a limited number of species, we hypothesized that *Acropora* gametes in the Indo-Pacific may preferentially fertilize conspecific gametes despite their compatibility with heterospecific gametes, leading to infrequent hybridization between potentially intercrossing species. In this study, F_1_ hybrids of *Acropora florida* and *A. intermedia* showed specific fertilization trends. For example, sperm had the ability to backcross with the parental species even in the presence of sperm from the parental species. Also, eggs of the hybrids produced from *A*. *florida* eggs and *A*. *intermedia* sperm (“FLOint”) exhibited self-fertilization. Since a low ratio of hybridization between *A. florida* and *A. intermedia* is predicted, the population size of hybrids should be small. Therefore, self-fertilization would facilitate reproduction of the hybrid in nature, while remaining sperm could outcompete parental species sperm to backcross with eggs. Although we succeeded in breeding two colonies of hybrids, it is reasonable to speculate that hybrids show a high tendency to choose the most efficient sexual reproduction tactics.

## Introduction

Hybridization is considered a mechanism for evolutionary innovation. Introgressive hybridization is caused by the repeated backcrossing of hybrids to the parental species^1^. Introgressive hybridization results in new gene combinations, leading to transgressive phenotypes^2,3^. Extensive hybridization is associated with the rapid diversification of species^4^. Moreover, hybrids can potentially occupy new habitats, differentiating them from the parental species^5^. Implications of hybridization for adaptation and hybrid fitness have been suggested^6–8^, but the ways in which introgression occurs in nature are still unknown.

In the Indo-Pacific, the reef-building coral *Acropora* spp. is species rich (> 110 species)^9^, and there is a potential relationship between hybridization and high species diversity^8^. For example, tabular species such as *A. hyacinthus* can form species complexes^9,10^, and gene flow among such species complexes occurs in a complex manner^11^. In addition, intermediate morphologies among intercrossing species imply that admixture events are associated with morphological diversity and similarity^10,12,13^. Morphological similarity is associated with hybridization/introgression in Caribbean^14–17^ and Indo-Pacific *Acropora*^8,12^. In both the Indo-Pacific and Caribbean, co-occurrence of spawning times/dates and gamete compatibility is related to introgression^18–20^. For repeated hybridization events among intercrossing species, the backcrossing of F_1_ hybrids to the parental species must occur, but the reproduction of F_1_ hybrids has not yet been fully investigated in the Southern Japanese Indo-Pacific, which is a hybrid hotspot area with high species richness of the coral *Acropora*^8,9^.

For introgression between two species, F_1_ hybrids from the two species must backcross with the parental species. However, such reproductive strategies, including the fertilization mechanisms of F_1_ hybrids in the Indo-Pacific, have not been fully elucidated, because Indo-Pacific hybrids other than *A. florida* and *A. intermedia*^21^ have not been successfully raised to their spawning age of approximately 7 years^21^. In nature, repeated hybridization between parental species arising from the backcrossing of F_1_ hybrids has been demonstrated^18^ in *A. prolifera*, the hybrid of two species inhabiting the Caribbean^14^. Therefore, although the importance of hybridization in the species-rich Indo-Pacific reef-building coral *Acropora* has been posited, there is no evidence showing how hybrids reproduce at the gametic level, or how they backcross and/or mate with each other.

Although introgression in the coral *Acropora* has been shown^7,8,11,22^, how F_1_ hybrids backcross with the parental species remains unknown. Our previous study showed that sperm of the F_1_ hybrids named “FLOint” and “INTflo”, bred from *A. florida* and *A. intermedia*, are compatible with eggs of the parental species^21^. In addition, eggs of the hybrid FLOint, raised from *A. florida* eggs and *A. intermedia* sperm, showed high selfing^21^. Ecologically, mating opportunities within F_1_ hybrids are more limited than are backcrossing opportunities with the parental species due to smaller numbers of F_1_ hybrid colonies. For hybrids to backcross with the parental species, the eggs of the parental species must accept hybrid sperm but tentatively prefer to mate with conspecific sperm in the presence of both conspecific and heterospecific sperm^23^. To clarify how gametes of the F_1_ hybrids mate, we examined whether hybrid sperm can outcompete parental species sperm, and whether hybrid FLOint eggs always show selfing. Using the results of these analyses, we show fertilization trends of the gametes of F_1_ hybrids (*A. florida* and *A. intermedia*) in the Indo-Pacific that lead to backcrossed and F_2_ generations of hybrids.

## Materials and methods

### Coral collection

In 2016, *Acropora florida* fragments from six colonies were collected from Majanohama, Akajima (Aka Island), Japan (26° 120 N, 127° 170 E), and two F_1_ hybrid colonies (INTflo: *A. intermedia* eggs × *A. florida* sperm; FLOint: *A. florida* eggs × *A. intermedia* sperm) were kept at Aka Island port. In 2016, we detected no *A. intermedia* with mature eggs, and thus *A. intermedia* spawning was supposed to have occurred during the previous full moon. In 2017, the hybrids (FLOint and INTflo) were used in experiments^12,21^, and two F_1_ hybrids, measuring approximately 30–50 cm, were transferred to Sesoko Station from Aka Island and maintained in an aquarium tank. In 2017, fragments from seven colonies of *A. florida* and 12 colonies of *A. intermedia* were collected from Sesoko Island (26° 37 N, 127° 51 E). All colonies and fragments were kept in a running seawater tank at the Akajima Marine Science Laboratory in 2016 or Sesoko Station at the University of the Ryukyus in 2017 until 1–5 days before their predicted spawning date.

### Spawning observation and gamete collection

Corals were observed at 20:30 from 5 days before their predicted spawning date on a full moon. When bundles were observed at the mouth of each polyp, the corals were transferred to a tank filled with seawater, and the time of spawning was recorded. The bundles were collected from the colony using plastic pipettes (Table 1), and gametes were separated into sperm and eggs using 100-µm plankton mesh, following Morita, et al.^24^. The sperm concentration of the isolated spermatozoa was determined using a hemocytometer. The final sperm concentrations were adjusted to approximately 10^4^, 10^5^, or 10^6^ sperm/mL for use in the subsequent crossing experiments.

**Table 1:** Spawning time of the coral *Acropora florida*,* A. intermedia*, and hybrid (FLOint, INTflo) in 2016 at Aka Island and 2017 at Sesoko Island.

Date	Time	Species	Colony names for fertilization experiments	
**2016 Aka**				
June 19th	21:40	*A. florida*	*f74	Full moon:June 20th
	21:50	*A. florida*	*f1,f15	
	22:00	*A. florida*, FLOint	*f11, *f13	
**2017 Sesoko**				
June 6th	22:08	*A. intermedia*	*i5, *i72	Full moon:June 9th
	22:09	INTflo		
	22:12	*A. intermedia*	*i2	
	22:13	*A. intermedia*	*i75	
June 16th	21:29	FLOint		
	21:35	INTflo		
July 7th	22:10	*A. florida*	f77	Full moon:July 9th
July 8th	21:56	*A. florida*	f77, f79	
July 11th	22:01	*A. florida*	f72	

*Indicate colonies used for crossing experiments.

### Crossing experiments and paternity tests

In the crossing experiments, approximately 200 eggs were transferred to 5 mL filtered seawater and 5 mL sperm suspension according to previously described methods^23,25^. The experiments were performed using *A. florida* and FLOint hybrid gametes on 19 June 2016 at Aka Island, and *A. intermedia* and INTflo hybrid gametes on 6 June 2017 at Sesoko Island. The spawning dates of *A. florida* and the two hybrids (INTflo and FLOint) did not coincide in 2017; INTflo spawned on 6 and 16 June and FLOint on 16 June, but *A. florida* spawned on 7, 8, and 11 July. Therefore, crossing experiments using FLOint and *A. florida* were not conducted.

For the crossing experiments, sperm concentrations were 10^4^, 10^5^, and 10^6^ sperm/mL for the sperm non-choice and sperm choice tests (fertilization trials in the presence of both parental species and hybrid sperm). In the crossing experiments, only colonies that spawned on the same day were used. *A. florida* and the FLOint hybrid were used in 2016, and INTflo and *A. intermedia* were used in 2017 (Table 1, Supplementary Table 1). The fertilization ratio was recorded based on whether or not eggs showed cell division within 2–4 h after mating at a temperature of 29 °C. Larvae from 3 to 4 days after fertilization were preserved in 99.5% ethanol and used for paternity tests. Paternity tests were performed to confirm which sperm fertilized the egg in the sperm choice test. DNA was extracted from the larvae, and microsatellite analysis was performed using the extracted DNA as a template with the markers 11745m3 and 11401m4^26^, according to Kitanobo et al.^23^. For each marker, fewer than two alleles were detected in the hybrids, and the same alleles were consistently detected from sperm and tissues, suggesting that the hybrids were not chimera (Supplementary data 1). Some analyses were conducted using acrylamide gel electrophoresis and others using fragment analysis with the ABI 3130xl or 3730xl DNA Sequencer (Applied Biosystems, Waltham, MA, USA). Microsatellite Analysis v1.0 (Applied Biosystems) software (https://www.thermofisher.com/order/catalog/product/4381867) was used to score the sizes.

### Statistical analyses

We conducted Tukey’s honestly significant difference (HSD) tests to evaluate differences in multiple comparisons. Welch’s two-sample *t*-tests were used to confirm differences in the fertilization ratio when using heterospecific sperm. All statistical analyses were performed using R version 4.01^27^.

### Ethical approval

All applicable international, national, and/or institutional guidelines for sampling, care, and experimental use of organisms for the study have been followed, and all necessary approvals have been obtained (No. 31-30).

## Results

### F_1_ hybrid spawning times and dates

Hybrids (FLOint; one colony) and *A. florida* (five colonies) released gametes in June 19–21, 2016 on Aka Island. *A. intermedia* did not release gametes synchronously with the hybrids. *A. intermedia* with mature eggs were not found, and it is likely that most *A. intermedia* had already spawned around the full moon at the end of May (Table 1). In 2017, spawning of the hybrid INTflo and *A. intermedia* coincided, but *A. intermedia*, *A. florida*, and the hybrid FLOint did not spawn on the same date (Table 1). Most colonies of *A. florida* spawned in July rather than June.

### Percentage of eggs fertilized in crosses among conspecifics

Crossing experiments using hybrids and parental species were conducted using *A. florida* and FLOint hybrid gametes on 19 June 2016, and *A. intermedia* and INTflo hybrid gametes on 6 June 2017. Fertilization ratios between the parental species were low in *A. florida* (< 11%) (Table S1). In contrast, fertilization ratios among *A. intermedia* were high under both low and adequate sperm concentrations, but the colony (I3_i2) had very low fertility ratios (Table S1).

### Inherent selfing and backcrossing of F_1_ hybrid eggs

Self-fertilization of the INTflo and FLOint gametes was examined. INTflo gametes did not show self-fertilization in the presence of low (10^4^ sperm/mL) to high (10^6^ sperm/mL) sperm concentrations (Table S1). By contrast, as in our previous study^21^, the eggs of FLOint showed high selfing ratios of 76%, 89%, and 76% under low, moderate and high sperm concentrations, respectively (10^4^, 10^5^, and 10^6^ sperm/mL) (Table S1).

To investigate the inherent ability of hybrid eggs to backcross with the sperm of the mother species, *A. florida* or *A. intermedia*, gametic compatibility was examined. Hybrid eggs showed a high ratio of fertilization to the sperm of the mother species; INTflo crossed with the sperm of *A. intermedia*, and FLOint showed a high ratio of fertilization to *A. florida* sperm (Fig. S1). Moreover, there was no significant difference in fertilization ratio among the different sperm concentrations (10^4^, 10^5^, and 10^6^ sperm/mL) for each hybrid (FLOint eggs × *A. florida* sperm: Tukey HSD P > 0.05, INTflo eggs × *A. intermedia* sperm: Tukey HSD P > 0.05).

**Figure 1:**
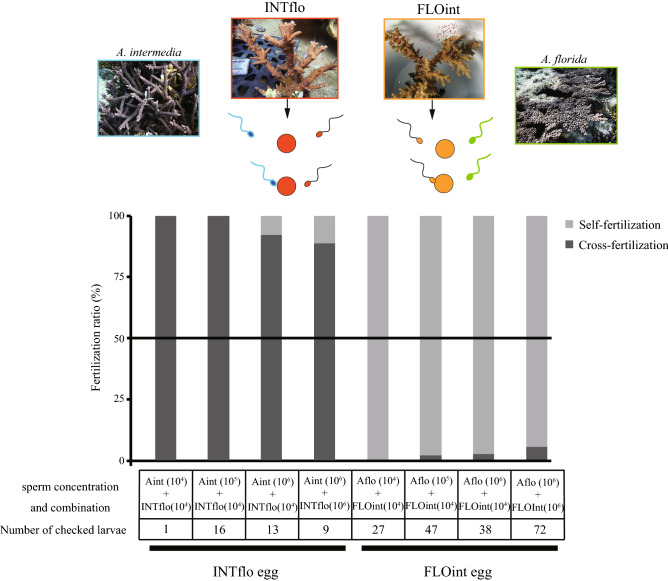
Backcrossing or self-fertilization of hybrid eggs. Sperm choice experiments using hybrid eggs are shown. INTflo and FLOint eggs showed different features: INTflo eggs backcrossed with the sperm of the mother species, whereas FLOint eggs self-fertilized as reported in Isomura et al.^21^. Light grey bars indicate the fertilization ratio of hybrid sperm (self-fertilization), and the dark grey bars indicate the sperm of the parental species. Microsatellites were checked using electrophoresis or Microsatellite Analysis v1.0 (Applied Biosystems) software (https://www.thermofisher.com/order/catalog/product/4381867).

Since high self-fertilization was observed for the eggs of FLOint in this and our previous study^21^, we performed paternity testing to determine whether FLOint eggs can be fertilized by *A. florida* sperm in sperm choice experiments (fertilization trials in the presence of both parental species and hybrid sperm). The results showed that most eggs were self-fertilized in the presence of both *A. florida* sperm and FLOint sperm (Fig. 1). On the other hand, most INTflo hybrid eggs backcrossed with *A. intermedia* sperm in the presence of INTflo sperm (Fig. 1).

### High compatibility of F_1_ hybrid sperm and eggs of the parental species

INTflo and FLOint sperm both backcrossed with eggs of the maternal parent of each hybrid (Fig. S2). For *A. florida* eggs, the ratio of fertilization to conspecific sperm was much lower (Table S1) than in previous trials, such as that conducted in 2015^21^, although the reason for this is unclear. For *A. intermedia* eggs, the fertilization ratio did not change with sperm concentration (*A. intermedia* eggs × INTflo sperm: Tukey HSD P > 0.05). As in *A. intermedia*, the fertilization ratio of *A. florida* eggs also did not significantly differ with sperm concentration (*A. florida* eggs × FLOint sperm: Tukey HSD P > 0.05).

### Can F_1_ hybrid sperm compete with conspecific sperm to backcross with eggs of the maternal species?

To examine backcrossing and self-fertilization ratios in the presence of parental species and hybrid sperm (*A. intermedia* and INTflo sperm or *A. florida* and FLOint sperm, respectively), paternity tests were performed. Fertilization ratios in the sperm choice experiments were not significantly different among combinations (Fig. S3; INTflo eggs × INTflo sperm and *A. intermedia* sperm: Tukey HSD P > 0.05, *A. intermedia* eggs × INTflo sperm and *A. intermedia* sperm: Tukey HSD P > 0.05).

**Figure 2:**
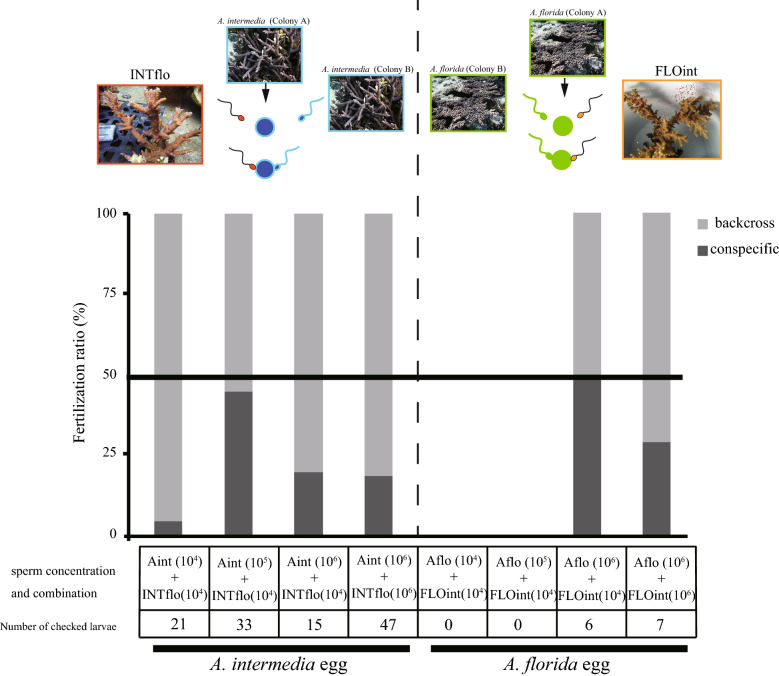
Backcrossing of hybrid sperm with parental species. Sperm choice experiments using eggs of the parental species are shown. Light grey bars indicate the fertilization ratio of parental species sperm, and white bars indicate the sperm of the hybrid species. Microsatellites were checked using electrophoresis or Microsatellite Analysis v1.0 (Applied Biosystems) software (https://www.thermofisher.com/order/catalog/product/4381867).

Eggs of the parental species (*A. florida* and *A. intermedia*) were fertilized by hybrid sperm in the presence of conspecific sperm. In case of INTflo sperm backcrossing, the hybrid sperm fertilized *A. intermedia* eggs independent of the sperm concentration of the parent species (Fig. 2, Tukey HSD P > 0.05) or hybrid sperm concentration (Tukey HSD P > 0.05). FLOint sperm also backcrossed with parent species *A. florida* eggs independent of hybrid sperm concentration (Fig. 2, *A. florida* egg × *A. florida* sperm and FLOint sperm; T-test, mt = − 0.1, df = 1.78, P > 0.05).

## Discussion

In our study, the F_1_ hybrid FLOint showed adequate fertilization patterns for reproduction, with high rates of backcrossing to the parental species and most of the eggs showing selfing. Our results showed that hybridization may arise when colony numbers decline due to heavy bleaching events. For example, *A. florida* eggs could hybridize with *A. intermedia* sperm at low sperm concentrations (FLOint)^23^. Conversely, *A. intermedia* eggs were preferentially fertilized by conspecific sperm when exposed to both *A. intermedia* and *A. florida* sperm^23^. Since the recovery of reefs after heavy bleaching often takes more than 10 years, and reef species composition changes frequently^28,29^, the post-bleaching F_2_ generation is predicted that they need to reproduce within lower number of colonies. In contrast to the Caribbean hybrid A*. prolifera* (< 25% selfing)^14^, FLOint showed more than 95% selfing (Fig. 1). Six to fourteen eggs and 10^6^ sperm are packed into *Acropora* gamete bundles^30,31^; thus sperm not involved in the self-fertilization of bundled eggs can mate with other unfertilized eggs. However, for backcrossing, spawning synchronicities of the hybrids FLOint and *A. intermedia* and INTflo and *A. florida* were not observed. Therefore, relationships between spawning synchronism and backcrossing with parental species still need to be clarified.

Sperm from the hybrids FLOint and INTflo potentially mate with parental species eggs when hybrid and parental species sperm compete. Sperm choice experiments in this study showed that the competencies of FLOint and INTflo sperm were high enough to outcompete parental sperm even when the number of hybrid sperm was far lower than that of the parental species sperm (Fig. 2). To support this result, repeated backcrossing is suggested in Caribbean *Acropora*^18^. In addition, there were fewer than two alleles of microsatellites, indicating polyploidy of the hybrids involving two chromosomes, unlike that reported in a previous study^32^. Therefore, fusion of the parental species is predicted to occur, but as the two parental species *A. florida* and *A. intermedia* are morphologically distinct, lineage fusion does not occur extensively at present.

From the present study, interspecific hybridization and introgression are suggested, but molecular based analyses for examining admixture events between the parental species are needed. From our preliminary SNP-based analyses, gene flow occurred among intercrossing species showing spawning synchronicity and high gamete compatibility (Kitanobo et al., unpublished data). However, the detection of hybridization is influenced by methodological differences^10^, and hybrid lineages are rare (five species)^8^. Moreover, integrative approaches from breeding trials and morphological and molecular based analyses indicate that morphologically distinguishable species can be reproductively isolated and can evolve independently^10^. Contrary to a previous study^33^, tabular species do not cross with other morphospecies^10^, but *A. florida* and *A. intermedia* gametes show high rates of intercrossing. In addition, gametes showed specific fertilization patterns according to sperm concentration, and the patterns of the F_1_ hybrids also indicate that backcrossing is highly probable. However, it seems difficult to distinguish between hybridization and incomplete lineage sorting if an admixture event occurred in the early speciated *Acropora* (< 6 Ma)^34^.

From our study, a slight admixture event between *A. florida* and *A. intermedia* may be ongoing, but detailed comprehensive studies involving other intercrossing species are needed. In this study, we focused on only two intercrossing species, *A. florida* and *A. intermedia*, but *A. intermedia* shows high rates of crossing with other sympatric and synchronous spawning species^35^. As discussed above, the morphologically distinct species of *A. florida* and *A. intermedia* show slight differences in spawning times and dates (Table 1); thus, these two species are tentatively at lower risk of hybridization. *A. intermedia* and other intercrossing species such as *A. gemmifera* are more likely to hybridize due to their overlapping spawning times^36^.

**Figure 3:**
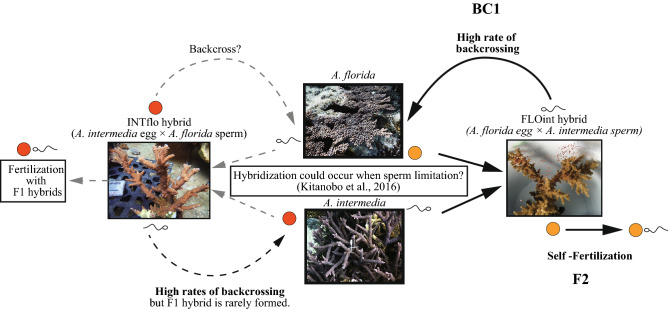
Schema of F1 hybrid reproduction. Flow from *A. intermedia* and *A. florida*, F1 hybrids, to reproduction of F1 hybrids. According to our studies, hybridization can occur when the sperm concentration is low, and an F1 hybrid (FLOint) can backcross or reproduce asexually. Bold lines indicate probable pathways, which are supported by this and our previous studies; faint grey lines indicate pathways that may not occur because of the low probability of hybridization.

Our study also shows that delimitation of species is suspected in the morphologically distinct intercrossing species *A. florida* and *A. intermedia*. Although our study used limited numbers of hybrids, the sperm competency of F_1_ hybrids was sufficient to provide opportunities for mating with parental species (backcrossing) and selfing in FLOint. This would be beneficial to the production of the F_2_ generation in cases of solitary spawning due to reduced spawning synchronicity or a reduced number of colonies of the parental species (Fig. 3). These features are congruent with high rates of introgression events under past climate changes^22,37^, and hybrid hotspots are located at biogeographic borders including Southern Japan^8^. Although the unique fertilization patterns of F_1_ hybrids are potentially not involved in ongoing hybridization, they may be a footprint of the past hybridization of ancestral species, and these results can be used to understand the complex history of the coral *Acropora*.

## Supplementary Information

Supplementary Data.

Supplementary Figure 1.

Supplementary Figure 2.

Supplementary Figure 3.

Supplementary Table.

## References

[1] Harrison RG, Larson EL (2014). Hybridization, introgression, and the nature of species boundaries. J. Hered..

[2] Seehausen O (2004). Hybridization and adaptive radiation. Trends Ecol. Evol..

[3] Mallet J (2007). Hybrid speciation. Nature.

[4] Wiens JJ, Engstrom TN, Chippindale PT (2006). Rapid diversification, incomplete isolation, and the "speciation clock" in North American salamanders (Genus plethodon): Testing the hybrid swarm hypothesis of rapid radiation. Evolution.

[5] Rieseberg LH (2003). Major ecological transitions in wild sunflowers facilitated by hybridization. Science.

[6] Chan WY, Hoffmann AA, van Oppen MJH (2019). Hybridization as a conservation management tool. Conserv. Lett..

[7] Richards ZT, Hobbs JPA (2015). Hybridisation on coral reefs and the conservation of evolutionary novelty. Curr. Zool..

[8] Hobbs J-PA (2021). Hybridisation and the evolution of coral reef biodiversity. Coral Reefs.

[9] Wallace CC (1999). Staghorn Corals of the World: A Revision of the Genus Acropora.

[10] Ramirez-Portilla C (2022). Solving the coral species delimitation conundrum. Syst. Biol..

[11] Ladner JT, Palumbi SR (2012). Extensive sympatry, cryptic diversity and introgression throughout the geographic distribution of two coral species complexes. Mol. Ecol..

[12] Fukami H, Iwao K, Kumagai NH, Morita M, Isomura N (2019). Maternal inheritance of F1 hybrid morphology and colony shape in the coral genus *Acropora*. PeerJ.

[13] Wolstenholme JK (2004). Temporal reproductive isolation and gametic compatibility are evolutionary mechanisms in the *Acropora humilis* species group (Cnidaria; Scleractinia). Mar. Biol..

[14] Vollmer SV, Palumbi SR (2002). Hybridization and the evolution of reef coral diversity. Science.

[15] Palumbi SR, Vollmer S, Romano S, Oliver T, Ladner J (2012). The role of genes in understanding the evolutionary ecology of reef building corals. Evol. Ecol..

[16] Kitchen SA (2019). Genomic variants among threatened *Acropora* corals. G3 (Bethesda).

[17] Kitchen SA (2020). STAGdb: A 30K SNP genotyping array and science gateway for *Acropora* corals and their dinoflagellate symbionts. Sci. Rep. Uk.

[18] Nylander-Asplin HF, Hill RL, Doerr JC, Greer L, Fogarty ND (2021). Population dynamics and genotypic richness of threatened *Acropora* species and their hybrid in the US Virgin Islands. Coral Reefs.

[19] Van Oppen MJ, Willis BL, Van Rheede T, Miller DJ (2002). Spawning times, reproductive compatibilities and genetic structuring in the *Acropora aspera* group: Evidence for natural hybridization and semi-permeable species boundaries in corals. Mol. Ecol..

[20] Fogarty ND, Vollmer SV, Levitan DR (2012). Weak prezygotic isolating mechanisms in threatened Caribbean *Acropora* corals. PLoS One.

[21] Isomura N, Iwao K, Morita M, Fukami H (2016). Spawning and fertility of F1 hybrids of the coral genus *Acropora* in the Indo-Pacific. Coral Reefs.

[22] Mao YF, Economo EP, Satoh N (2018). The roles of introgression and climate change in the rise to dominance of *Acropora* corals. Curr. Biol..

[23] Kitanobo S, Isomura N, Fukami H, Iwao K, Morita M (2016). The reef-building coral Acropora conditionally hybridize under sperm limitation. Biol. Lett..

[24] Morita M (2006). Eggs regulate sperm flagellar motility initiation, chemotaxis and inhibition in the coral *Acropora digitifera*, *A. gemmifera* and *A. tenuis*. J. Exp. Biol..

[25] Ohki S, Kowalski RK, Kitanobo S, Morita M (2015). Changes in spawning time led to the speciation of the broadcast spawning corals *Acropora digitifera* and the cryptic species *Acropora* sp. 1 with similar gamete-recognition systems. Coral Reefs.

[26] Shinzato C (2014). Development of novel, cross-species microsatellite markers for *Acropor*a corals using next-generation sequencing technology. Front. Mar. Sci..

[27] *R: A Language and Environment for Statistical Computing* (R Foundation for Statistical Computing, 2020).

[28] Muko S, Suzuki G, Saito M, Nakamura T, Nadaoka K (2019). Transitions in coral communities over 17 years in the Sekisei Lagoon and adjacent reef areas in Okinawa, Japan. Ecol. Res..

[29] van Woesik R, Sakai K, Ganase A, Loya Y (2011). Revisiting the winners and the losers a decade after coral bleaching. Mar. Ecol. Prog. Ser..

[30] Furukawa M, Ohki S, Kitanobo S, Fukami H, Morita M (2020). Differences in spawning time drive cryptic speciation in the coral *Acropora divaricata*. Mar. Biol..

[31] Wallace C (1985). Reproduction, recruitment and fragmentation in nine sympatric species of the coral genus *Acropora*. Mar. Biol..

[32] Kenyon JC (1997). Models of reticulate evolution in the coral genus *Acropora* based on chromosome numbers: Parallels with plants. Evolution.

[33] Willis BL, Babcock RC, Harrison PL, Wallace CC (1997). Experimental hybridization and breeding incompatibilities within the mating systems of mass spawning reef corals. Coral Reefs.

[34] Fukami H, Omori M, Hatta M (2000). Phylogenetic relationships in the coral family acroporidae, reassessed by inference from mitochondrial genes. Zool. Sci..

[35] Hatta M (1999). Reproductive and genetic evidence for a reticulate evolutionary history of mass-spawning corals. Mol. Biol. Evol..

[36] Baird AH (2021). An Indo-Pacific coral spawning database. Sci. Data.

[37] Mao YF (2019). Genomic insights into hybridization of reef corals. Coral Reefs.

